# Correction: Analysis of a new negevirus-like sequence from *Bemisia tabaci* unveils a potential new taxon linking nelorpi- and centiviruses

**DOI:** 10.1371/journal.pone.0327910

**Published:** 2025-07-09

**Authors:** Diego F. Quito-Avila, Edison Reyes-Proaño, Gerardo Armijos-Capa, Ricardo I. Alcalá Briseño, Robert Alvarez, Francisco J. Flores

The sixth author’s name is spelled incorrectly. The correct name is: Francisco J. Flores. The correct citation is: Quito-Avila DF, Reyes-Proaño E, Armijos-Capa G, Alcalá Briseño RI, Alvarez R, Flores FJ (2024) Analysis of a new negevirus-like sequence from Bemisia tabaci unveils a potential new taxon linking nelorpi- and centiviruses. PLoS ONE 19(5): e0303838. https://doi.org/10.1371/journal.pone.0303838
