# Supplementary figures and images for: Analysis of a new negevirus-like sequence from Bemisia tabaci unveils a potential new taxon linking nelorpi- and centiviruses
Source: PLoS One. 2024 May 16;19(5):e0303838. doi: 10.1371/journal.pone.0303838 (PMC11098327; doi:10.1371/journal.pone.0303838)

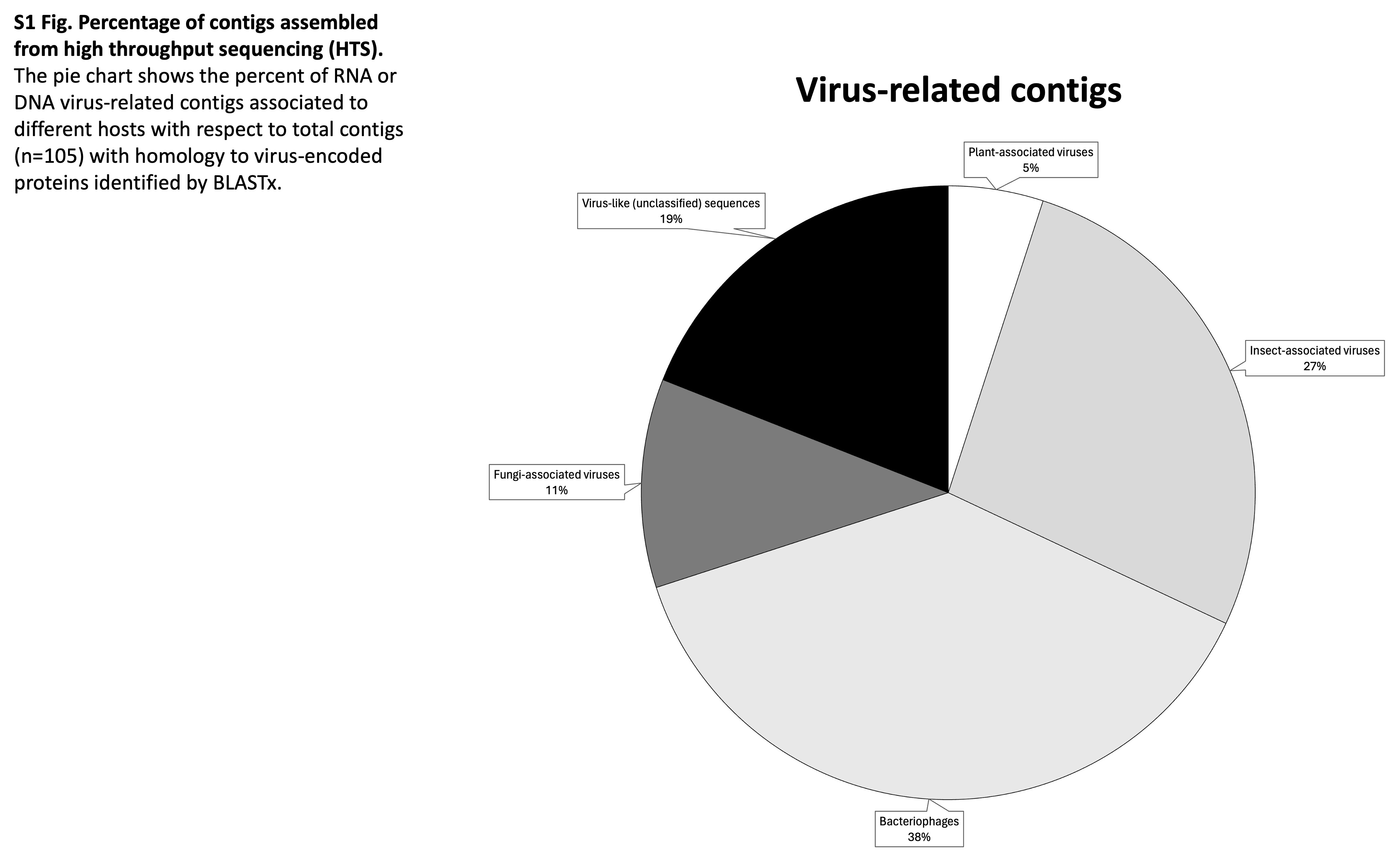

Supplement: S1 Fig — The pie chart shows the percent of RNA or DNA virus-related contigs associated to different hosts with respect to total contigs (n = 105) with homology to virus-encoded proteins identified by BLASTx. (JPG) [file pone.0303838.s001.jpg]
