# Supplementary material for: Analysis of a new negevirus-like sequence from Bemisia tabaci unveils a potential new taxon linking nelorpi- and centiviruses
Source: PLoS One. 2024 May 16;19(5):e0303838. doi: 10.1371/journal.pone.0303838 (PMC11098327; doi:10.1371/journal.pone.0303838)
Supplement: S1 Table — The following PCR parameters are recommended for all the primer sets used to re-sequence the genome: 96°C x 4 min, 40 cycles of 96°C x 1 min, 55°C x 30 sec, and 72°C x 1.5 min, a final additional extension at 72°C x 5 min. For rapid-amplification of cDNA-ends (RACE) refer to manufacturer instructions. (DOCX) [file pone.0303838.s002.docx]

**S1 Table.** List of primers used for resequencing the whitefly negevirus 1 (WfNgV1) genome. The following PCR parameters are recommended for all the primer sets used to re-sequence the genome: 96°C x 4 min, 40 cycles of 96°C x 1 min, 55°C x 30 sec, and 72°C x 1.5 min, a final additional extension at 72°C x 5 min. For rapid-amplification of cDNA-ends (RACE) refer to manufacturer instructions.

| **Primer name** | **Sequence 5′ to 3′** | **Target genome^*^ position**  **(amplicon size nt)** |
| --- | --- | --- |
| 1Race-5’end-1 | CGAGCATAAGCTGTACAGATTGC | 201^#^ |
| 1Race-5’end-2 | CTGAAAGACGGATAGTGCGTTGT | 372^#^ |
| WfNgV1-CovF1 | CCCTTCTTCTTGTGATTCATATGC | 15-1,330  (1,316) |
| WfNgV1-CovR1 | GCAGGCACAATAAGACGGATGG |  |
| WfNgV1-CovF2 | CCTCTCGCATTTTGACCAACGA | 1,198-2,393  (1,196) |
| WfNgV1-CovR2 | GTCCATCTCCAAGCAAGGGTCTA |  |
| WfNgV1-CovF3 | GCATTCATACACCTGCCGATTGC | 2,257-3,589  (1,333) |
| WfNgV1-CovR3 | CGAGGTCCAATAGTAGAGCGGTT |  |
| WfNgV1-CovF4 | GCAGGGATTTAAGGATCTGCGGT | 3,460-4,776  (1,317) |
| WfNgV1-CovR4 | CGCTATGTGCTCTATTTGTGGAG |  |
| WfNgV1-CovF5 | GTACCTGCTCCTATCACCTCTA | 4632-5907  (1,276) |
| WfNgV1-CovR5 | CGAACAAGGCTGCATGATCGAAG |  |
| WfNgV1-CovF6 | GTTCCTGTCCACAAGCTCTCACT | 5,769-7,051  (1,283) |
| WfNgV1-CovR6 | CCAGGAAAGGTTGAAGAATCACC |  |
| WfNgV1-CovF7 | GGGATAACTCTCATATTTCGACCG | 6,949-8,370  (1422) |
| WfNgV1-CovR7 | CGGATGCAATCGCATCCGATA |  |
| WfNgV1-CovF8 | GCGTGTTTTGAGTCTGATCACG | 8,318-9,659  (1,342) |
| WfNgV1-CovR8 | GAGCCACATTCCTTCTGCGTA |  |
| WfNgV1-CovF9 | CATCCACCTCACAATATGATTGCA | 9,614-10,612  (999) |
| WfNgV1-CovR9 | GTGTGCTGCTCAATGCAAACAAC |  |
| WfNgV1-CovF10 | CGCTTACACCAGTAATGTTGTTGC | 10,534-11,726  (1,193) |
| WfNgV1-CovR10 | GGTATCCTGGTCGTAATGGTCAC |  |
| 3Race-3’end-1 | CGCTAATACAGAATCACACGCG | 11,402^#^ |
| 3Race-3’end-2 | GCTAATCGTCACACACAACACGC | 11,610^#^ |

^#^Denotes genome position of the primer, but the amplificon size is dependent on the poly A length where RACE oligo-dT-anchored primers laid down.

*Primer sets recommended for routine detection
