# Supplementary material for: Analysis of a new negevirus-like sequence from Bemisia tabaci unveils a potential new taxon linking nelorpi- and centiviruses
Source: PLoS One. 2024 May 16;19(5):e0303838. doi: 10.1371/journal.pone.0303838 (PMC11098327; doi:10.1371/journal.pone.0303838)
Supplement: S2 Table — n.a. denotes no homologue available. (DOCX) [file pone.0303838.s003.docx]

**S2 Table.** Virus names and accession numbers for amino acid sequences used in the alignments for Figure 2. n.a. denotes no homologue available.

|  |  | **Accession numbers** | | | |
| --- | --- | --- | --- | --- | --- |
| **Virus** | **Abbreviation** | **RdRp** | **Glycoprotein** | **SP24** | **Hypothetical ORF 4 protein** |
| Whitefly negevirus 1 | WfNgV1 | WRT26033 | WRT26034 | WRT26035 | WRT26036 |
| Bemisia tabaci negevirus 1 | BtNeV1 | QWC36478 | QWC36479 | QWC36480 | QWC36479.1 |
| Negev virus | NEGV | UZH43557 | BAR91506 | UZH43559 | n.a. |
| Aphis glycines virus 3 | ApGlV3 | ASH89118 | ASH89119 | ASH89121 | n.a. |
| Beihai barnacle virus 2 | BBV2 | YP_009333216 | YP_009333217 | YP_009333218 | n.a. |
| Citrus leprosis virus | CiLV | ABG33779 | n.a. | ABD59465 | n.a. |
| Blueberry necrotic ring blotch virus | BNRBV | AGI44298 | n.a. | YP_004901704 | n.a. |
| Hibiscus green spot virus 2 | HGSV2 | WMQ58794 | n.a. | YP_004928121 | n.a. |
| Tetranychus urticae kitavirus | TuKV | MN204568 | n.a. | MN204568 | n.a. |
| Red mite virga-like virus 1 | RMVLV1 | UQT02528 | n.a. | UQT02530 | n.a. |
| Chronic bee paralysis virus | CBPV | ACO82548 | ASM62179.1 | ASM62180 | n.a. |
| Wuhan house centipede virus 1 | WHCV-1 | BBV14741 | BBV14742 | BBV14743 | n.a. |
| Bemisia tabaci nege-like virus 1 | BtNeLV-1 | QWC36482 | n.a. | n.a. | n.a. |
| Bemisia tabaci nege-like virus 2 | BtNeLV-2 | QWC36484 | n.a. | n.a. | n.a. |
| Bemisia tabaci nege-like virus 3 | BtNeLV-3 | QWC36485 | n.a. | n.a. | n.a. |
| Bustos virus | BUSV | BAU71147 | Not found | BAU71149 | n.a. |
| Drosophila melanogaster * | n.a. | n.a. | n.a. | ABC86319 | n.a. |
