# Supplementary material for: Analysis of a new negevirus-like sequence from Bemisia tabaci unveils a potential new taxon linking nelorpi- and centiviruses
Source: PLoS One. 2024 May 16;19(5):e0303838. doi: 10.1371/journal.pone.0303838 (PMC11098327; doi:10.1371/journal.pone.0303838)
Supplement: S3 Table — n.a. denotes no homologue available. (DOCX) [file pone.0303838.s004.docx]

**Supplementary Table S3.** Virus names and accession numbers for amino acid sequences corresponding to putative structural protein of 24 kDa (SP24) and RNA-dependent-RNA-polymerase (RdRp) used for phylogenetic analyses (Figure 3). n.a. denotes no homologue available.

|  |  | **Accession numbers** | |
| --- | --- | --- | --- |
| **Proposed or formal genus** | **Virus name** | **SP24** | **RdRp** |
| *Sandewavirus* | Goutanap virus | AIX97864 | AIX97862 |
|  | Dezidougou virus | AFI24671 | AFI24669 |
|  | Dezidougou virus | QIN93581 | QIN93579 |
|  | Bustos virus | BAU71149 | BAU71147 |
|  | Wallerfield virus | AQM55506 | AQM55504 |
|  | Santana virus | AFI24677 | AFI24675 |
|  | Tanay virus | YP_009028560 | AHX42602 |
|  | Biratnagar virus | YP_009351826 | AQM55290 |
|  | Feitosa virus (BeAr805514) | UZH43556 | UZH43554 |
|  | Feitosa virus (BeAr805503) | UZH43541 | UZH43539 |
|  | Wallerfield virus (BeAr805520) | UZH43565 | UZH43563 |
| *Nelorpivirus* | Negev like virus (BeAr805514) | UZH43559 | UZH43557 |
|  | Cordoba virus (BeAr805503) | n.a. | UZH43538 |
|  | Cordoba virus (EVG9-228B) | n.a. | AQM55306 |
|  | Negev like virus 174 | CRL87032 | CCV01575 |
|  | Negev virus | BAR91507 | BAR91505 |
|  | Culex Negev 730 like virus | AXQ04833 | AXQ04831 |
|  | West Accra virus | BBN20801 | BBN20799 |
|  | Castlerea virus | YP_009362300 | AQZ55393 |
|  | Manglie virus | UUG74165 | UUG74163 |
|  | Ngewotan negevirus | AFY98074 | AFY98072 |
|  | Daeseongdong virus 1 | YP_009182193 | YP_009182191 |
|  | Negevirus Nona 1 | BAS69362 | BAS69360 |
|  | Ying Kou virus | YP_009552741 | YP_009552739 |
|  | Piura virus | AQM55375 | AQM55498 |
|  | San Bernardo virus | YP_009351829 | YP_009351827 |
|  | Brejeira virus | AQM55486 | AQM55484 |
|  | Big Cypress virus | YP_009351823 | YP_009351821 |
|  | Loreto virus | YP_009351837 | YP_009351835 |
| *Centivirus* | Indomegoura indica nege-like virus 1 | QUE41596 | QUE41594 |
|  | Astegopteryx formosana nege-like virus 1 | URA30366 | URA30365 |
|  | Wuhan insect virus 8 | YP_009344996 | APG77763 |
|  | Wuhan house centipede virus 1 | BBV14743 | YP_009345002 |
|  | Barley aphid RNA virus 1 | BBV14747 | BBV14745 |
|  | Aphis glycines nege-like virus 1 iso AG1 | n.a. | UTQ79680 |
|  | Aphis glycines nege-like virus 1 iso-1 | UTQ79677 | UTQ79675 |
|  | Aphis glycines nege-like virus 1 iso ABC1 | n.a. | UTQ79653 |
|  | Hubei virga-like virus 4 | n.a. | APG77770 |
| *Aphiglyvirus* | Barley aphid RNA virus 3 | BBV14753 | WIM36807 |
|  | Wuhan insect virus 9 | YP_009345004 | BBV14751 |
|  | Aphis glycines virus 3 | ASH89121 | YP_009333216 |
| *Cilevirus* | Citrus leprosis virus C2 | WAW15953 | WAW15948 |
|  | Passion fruit green spot virus isolate_BJL1 | QFU28443 | QFU28437 |
|  | Passion fruit green spot virus Bz | QIH54362 | QIH54363 |
|  | Citrus leprosis virus | YP_009508076 | YP_009508070 |
|  | Hibiscus infecting cilevirus | ATW76028 | ATW76030 |
|  | Cytoplasmic citrus leprosis virus | ABD59465 | ABG33779 |
|  | Vinca ringspot virus | WHW95146 | WHW95142 |
|  | Ligustrum leprosis virus | YP_010840894 | YP_010840895 |
|  | Ligustrum chlorotic spot virus | YP_010840891 | YP_010840887 |
|  | Solanum violifolium ringspot virus | YP_010840886 | YP_010840882 |
|  | Pistachio virus Y | QPL17818 | QPL17819 |
|  | Hibiscus yellow blotch virus | YP_010840389 | YP_010840385 |
| *Higrevirus* | Pistachio virus X | QPL17809 | QPL17815 |
|  | Hibiscus green spot virus 2 | YP_004928121 | YP_004928118 |
|  | Higrevirus Waimanalo | WMQ58801 | WMQ58794 |
| *Blunervirus* | Tomato blunervirus 1 | YP_010840082 | YP_010840077 |
|  | Bluner-sp Camelia japonica | UYO08084 | UYO08081 |
|  | Tea plant necrotic ring blotch virus iso-Iran | UJR02103 | URQ09708 |
|  | Ailanthus crinkle leaf associated bluner-like | WCL16057 | WCL16051 |
|  | Poaceae liege blunervirus | WCJ13292 | WCJ13289 |
|  | Blueberry necrotic ring blotch virus | YP_004901704 | YP_004901700 |
| Unclassified *kitavirids* | Tetranychus_urticae_kitavirus | MN204568 | MN204568 |
|  | Varroa jacobsoni virus 4 | QKW94175 | MN204568 |
|  | Chrysanthemum kita-like virus | WIM36810 | QKW94174 |
| Unclassified *negevirids* | Frankliniella occidentalis associated negev-like virus 1 | n.a. | QNM37802 |
|  | Frankliniella occidentalis associated negev-like virus 2 | n.a. | QNM37799 |
|  | Frankliniella occidentalis associated negev-like virus 3 | n.a. | QNM37811 |
|  | Utsjoki negevirus 1 | UUV42190 | UUV42185 |
|  | Soybean thrips nege-like virus 1 | QQP18765 | QQP18763 |
|  | Beihai barnacle virus 2 | YP_009333218 | BBV14741 |
| Unclassified whitefly-associated negevirids | Bemisia tabaci negevirus 1 | QWC36480 | QWC36478.1 |
|  | Bemisia tabaci nege-like virus 1 | n.a. | QWC36482 |
|  | Bemisia tabaci nege-like virus 2 | n.a. | QWC36484 |
|  | Bemisia tabaci nege-like virus 3 | n.a. | QWC36485 |
|  | Whitefly negevirus 1 | WRT26035 | WRT26033 |
| *Chroparavirus* | Linepithema humile C virus 1 | UXD80036 | UXD80112 |
|  | Anopheline associated C virus | YP_009011229 | YP_009011225 |
|  | Wuhan insect virus 21 | BBV14767 | QQP18791 |
|  | Chronic bee paralysis virus | ASM62180 | ASM62175 |
| Other unclassified | Red mite virga-like virus 1 | UQT02530 | UQT02528 |
|  | Drosophila melanogaster associated SP24* | ABC86319 | n.a. |

*This SP24 homologue found in Drosophila melanogaster has only been hypothesized to belong to a virus.
