# Supplementary material for: Analysis of a new negevirus-like sequence from Bemisia tabaci unveils a potential new taxon linking nelorpi- and centiviruses
Source: PLoS One. 2024 May 16;19(5):e0303838. doi: 10.1371/journal.pone.0303838 (PMC11098327; doi:10.1371/journal.pone.0303838)
Supplement: Attachment — Submitted filename: Review_2024_PLOSONE_QuitoAvila.docx [file pone.0303838.s005.docx]

**Summary**

Whitefly negevirus 1 is a fascinating novel virus identified in an agriculturally significant host and falls within an evolutionarily interesting clade. The authors describe this novel virus in detail genomically and phylogenetically. The phylogenies in this paper also appear to link *Nelorpivirus* and *Centivirus*, hopefully bringing us a step to formally ratifying these clades. The methodology is reproducible and well-described overall. I have generally minor comments and queries throughout the manuscript as well as some suggestions for figure edits that should improve the ease of interpretation for a reader. These are all outlined below in line-by-line suggestions. Overall, this is a robust paper that is sure to be of interest to the readers of PLOS ONE, and will be particularly valuable to readers focused on arthropod-associated viruses or unique and divergent RNA virus clades.

**Comments**

1. **Line 93:** The data set is comprised of one library constructed from ten individual whiteflies. It would be helpful to explain why the samples were combined and to indicate at which stage samples were combined (i.e., before or after RNA extraction).

2. **Line 132:** It is unclear how many ambiguously aligned positions were removed. Can the authors provide the lengths of the original alignments and compare to the lengths of trimmed alignments?

3. **Line 174:** What were the percentage identity cut-offs for considering a contig to be viral? Were these contigs further analysed (phylogenetically for example) to confirm they were indeed viral? And how were host organisms assigned? This would greatly enhance the level of detail in the methods section.

4. **Line 174:** Does this data set of 105 contigs include RNA viruses or all viruses? This should be stated in the methods as well as the figure legend for Supplementary Figure 1.

5. **Figure 2 legend:** A description of Panel D appears to be missing in this figure legend.

6. **Line 257:** Please refer to the relevant figures earlier in each section for ease of understanding.

7. **Figure 2B:** The highlighted red valine residue and lack of position markings seems to imply the fourth conserved cysteine residue is not present and is instead a conserved valine residue. Consider marking the positions of interest and highlighting the fourth cysteine residues in position 114, perhaps by shading the column in grey as it is not universally conserved like positions 70, 84, and 90.

8. **Line 258:** What are the “additional conserved residues”? Cysteine?

9. **Line 265:** This section needs more references to the relevant figure, especially earlier on. It’s very difficult to follow the text without knowing which figure represents the described data.

10. **Line 267:** “(refer to the subsequent section)” This is confusing as it is unclear when reading what subsequent section is being referred to.

11. **Figure 2C:** It may be helpful to highlight the exact residues considered to be conserved here. Perhaps they can be bolded so it is clear, particularly in some of the less obviously conserved positions (e.g., in the first shaded column of Figure 2C, bold each occurrence of the letter D).

12. **Lines 271-289:** The entire section refers often to the amino acid compositions of different parts of the WfNgV1 genome. The significance of these observations are not discussed. I encourage the authors to expand on this idea in both the results and discussion sections so it is clear why the amino acid compositions matter.

13. **Line 285:** It may be helpful to put the single-letter code in brackets when a full amino acid name is written to assist those who do not have the abbreviations memorized. That will make it easier to quickly refer to the figure and identify the residue in question.

14. **Figure 3A:** It is interesting that the *Kitaviridae* and kita-like RdRp sequences in this tree are not monophyletic, in contrast to the other trees. What do the authors think of this observation?

15. **Figure 3:** The spacing in between certain branches is strange. Is this simply a visual artifact or are these large gaps intentionally added to signify something?

16. **Figure 3:** What do the branch lengths and scale bar represent? Number of amino acid changes per site? This information should be added to the figure legend.

17. **Figure 4:** The authors may want to reconsider the colours used in this figure (particularly panels C and D). The current palettes may be difficult for readers with reduced colour vision or those reading in black and white.

18. **Figure 4D:** As the scale is from 0 to 1, it may be better to have a single-colour scale, ie, eliminate the blue shades and represent 0 with white and 1 with dark red.

19. **Line 380:** Avoid referencing figures in the discussion, that should be restricted to results. Here, the results should be expanded on in the context of currently available literature.

20. **Line 385:** Low bootstrap support indicates low confidence in the relationships inferred from presented topology; other causes, such as poor alignment and conflicting evolutionary signals, must be acknowledged.

21. **Line 569:** I highly recommend representing these data differently, at least by making it a flat pie chart rather than 3D. Also, the colours are unfortunately completely indistinguishable in black and white and quite difficult to distinguish for those with colour blindeness. Please change the colour palette or consider labelling the slices directly with the relevant category.

22. **Line 581:** Please ensure formally classified taxa are formatted correctly (i.e., italicised) throughout the text and in this table.
